# *FiberO* for an automated quantitative analysis of fibers orientation and organization in biological fibrous tissues

**DOI:** 10.3389/fbioe.2024.1497837

**Published:** 2025-01-06

**Authors:** Asier Muñoz, Anxhela Docaj, Julen Fernandez, Alessandra Carriero

**Affiliations:** Department of Biomedical Engineering, The City College of New York, New York, NY, United States

**Keywords:** fiber, orientation, organization, continuity, fibrous tissue

## Abstract

Many biological fibrous tissues exhibit distinctive mechanical properties arising from their highly organized fibrous structure. In disease conditions, alterations in the primary components of these fibers, such as type I collagen molecules in bone, tendons, and ligaments, assembly into a disorganized fibers architecture generating a weak and/or brittle material. Being able to quantitatively assess the fibers orientation and organization in biological tissue may help improve our understanding of their contribution to the tissue and organ mechanical integrity, and assess disease progress and therapy effect. In this work, we present *FiberO*, a new open-source available software that automatically quantifies fibers orientation, by performing morphological image openings, and fibers organization within the tissue, by determining and plotting their continuity in groups. *FiberO* performance is here evaluated using second harmonic generation microscopy images of mouse bones and tendons as examples of biological fibrous tissues. *FiberO* outperformed *Directionality* and *OrientationJ*, two open-source plugins available in ImageJ, and *FiberFit* and *CT-FIRE,* in the calculation and plotting of fibers orientation in reference images with known fibers orientation. Additionally, *FiberO* is currently the sole software to date able to accurately track the continuity of aligned fibers, and it quantifies and displays the organized surface(s) in the tissue of interest. *FiberO* can be used in the wider engineering and science field to investigate the fibers orientation and organization of different natural and synthetic fibrous tissues.

## Introduction

Biological fibrous tissues are an incredible source of inspiration for new man-made materials. Their composite nature and complex hierarchical structure confer them ultimate material properties that are difficult to find in new materials. In particular, bone, the tissue constituting our skeleton, is a biological composite material made of a mineral phase, based on hydroxyapatite mineral crystals, embedded in a collagen protein mesh. The hierarchical arrangement of its primary components makes bone a strong and tough material (Muñoz et al., 2021). Tendons and ligaments are soft connective tissues able to sustain high tensile loads thanks to their collagenous hierarchical organization. Similarly to skin and blood vessels, which are also composed by elastin, they are energy store tissues able to regain their original shape after being loaded in elastic conditions. These biological fibrous tissues are a great source of inspiration for newly generated materials; yet we still do not know how exactly the hierarchical structure confers them their material properties. Therefore, knowing how their primary components are organized within the material, particularly their fibers arrangement, will help reveal how these biological materials develop their material properties, i.e., stiffness, strength and toughness. Indeed, despite a good amount of research conducted in the field, it is still unclear the link between the structural and compositional properties of fibers (in particular collagen and other fibrous proteins organization) in biological materials and their mechanical properties.

Imaging fibers structure and organization within the biological tissues can be very informative to (i) understand the biomechanical properties of the tissue, (ii) detect and study progression of disease, and (iii) assess efficacy of treatments. Particularly, second harmonic generation (SHG) microscopy, a derivative of multiphoton microscopy, has a unique ability to directly image collagen fibers structure in different tissues, including tendon, ligament, bone (Millard et al., 2003), and lung (Kottmann et al., 2015) tissue without the need for labeling with either fluorophores or fluorescent proteins (Williams et al., 2001; Zipfel et al., 2003). Type I collagen generally produces a very strong signal in both backward and forward SHG microscopy, whereas collagen type II, III, V, XI, XXIV and XXVII generate weaker and sometimes not sufficient signal (Yoshioka et al., 2022; Ranjit et al., 2015; Chen et al., 2012; Lilledahl et al., 2011). Apart from collagen, acto-myosin, microtubules and elastin have been previously imaged using SHG microscopy in their endogenous tissues, yet collagen type I signal remains the most efficient SHG source proteins with subsequently brightest signal compared to any other fibrous protein (Campagnola et al., 2002; Chu et al., 2004; Dombeck et al., 2003; Le et al., 2007; Mansfield et al., 2009). One of the principal advantages of SHG microscopy over many other imaging techniques is that it achieves a high level of penetration in both thick and dense tissue samples, allowing for visualization of the collagen assembly with no need for fixation. Additionally, live cell and tissue imaging is also possible since there is no excitation of molecules nor photobleaching of the tissue (Williams et al., 2001; Kim et al., 2000). SHG microscopy imaging allows for visualization of collagen protein assembly and organization at the sub-micrometer and micrometer scales (Campagnola et al., 2002; Chu et al., 2004). This particular imaging technique has its origin in the induction of intense laser radiation in tissue samples resulting in frequency doubling, i.e., the optical effect in which two incident photons combine and emit a single photon with visible light (Millard et al., 2003; Campagnola et al., 1999; Mohler et al., 2003; Roth and Freund, 1981).

In diseases or pathological conditions that are associated with fibers disorganization such as fibrosis (Kottmann et al., 2015; Parra et al., 2006; Rittié, 2017), cancers (Rittié, 2017; Cloos et al., 2003; Zunder et al., 2020), atherosclerosis (Le et al., 2007), cleft lip (Noor et al., 2022), or cases pertaining to connective tissues and disorders such as tendinopathy (Knapik and Pope, 2020), osteoarthritis (Yoshioka et al., 2022), fracture healing (Nair et al., 2022), skin damage (Mostaço-Guidolin et al., 2017), Paget’s disease (Cloos et al., 2003) or osteogenesis imperfecta (OI) (Shapiro et al., 2021), alterations of fibrous proteins orientations have been previously observed using polarized light and SHG microscopy. However, the inability to quantify their orientation and organization has left us with very interesting questions that researchers are trying to solve regarding the assessment of their direct contribution to their endogenous tissue’s mechanical properties. Similarly, tissue engineering research also sees a rising need for a better understanding of the link between fibers structure organization and tissue mechanics to create new materials with formidable mechanical performances (Kim et al., 2021).

The orientation of biological tissue fibers visualized with SHG microscopy has been previously quantified using a variety of computational methods. These include the 2D Fourier transform (Bueno et al., 2013; Sivaguru et al., 2010), Fourier transform followed by power spectral density determination (Bayan et al., 2009), Fourier transform combined with a Radon transform (McLean, 2015), edge detection technique (Hill et al., 2012), watershed (Koch et al., 2014), and wavelet transforms (Tilbury et al., 2014). Some of these methods require significant image pre-processing steps that can delete useful information or amplify the noise in images with poor signal-to-noise ratio (Bredfeldt et al., 2014). For instance, Fourier transform often requires extensive noise reduction, contrast enhancement and image scaling to improve the accuracy of frequency domain analysis (Singh and Mittal, 2014). Similarly, edge detection methods involve pre-processing steps like smoothing or thresholding to isolate relevant edges while the watershed algorithm requires careful image smoothing and segmentation, especially in cases where boundaries are unclear (Zhao and Xie, 2013). Others are based on computationally expensive image transformation techniques (Barbhuiya and Hemachandran, 2013; Deans, 2007). However, it is still challenging to automatically and quantitatively determine how these fibers are arranged within the tissue, and discern the regions of organized and disorganized fibers. These regions are usually visually inspected and manually separated in the images of interest (Hedjazi et al., 2022). Human perception can be appropriate for visually detecting well-organized fiber structures from images, such as those from SHG microscopy, but this would only be as far as a qualitative analysis, and it would fail at a quantitative assessment of such information, especially when the comparison between samples of different groups is necessary. Therefore, for an accurate evaluation of fibers organization in SHG microscopy images, a methodology that is based on a quantitative and automated analysis of fibers continuity is essential, and this has been the focus of this work.

The development of a reliable, open-source, quantitative approach that enhances our understanding of the fibers alignment and organization in biological tissues, such as bone, can help identify diseases, assess their progress and efficacy of treatments in the clinical community. Importantly, having an open-source tool that provides accurate results offers the opportunity to compare results in consistent manner using a standardize platform at no cost for the researchers worldwide. Here we present *FiberO*, a new quantitative and objective software that automatically identifies fibers, and quantifies their orientation and organization within the tissues, according to the fibers contiguous structure. The morphological opening applied to the SHG microscopy images used in this study allows the creation of maps depicting the collagen fibers orientation. Furthermore, the application of 8-neighbor connectivity of the angular data from the output images of the previous step enables to trace tissue organization in terms of fibers continuity. This standardized and unbiased method for interpreting greyscale images of fibers, as those obtained from SHG microscopy images, can be used to support material characterization, clinical studies of biopsies, tissue engineering as well as any other analysis of fibrous tissues.

## Methods

### Images of fibers within tissues: SHG microscopy images of bone and tendon

To demonstrate the value and performance of *FiberO*, our new methodology, we considered SHG microscopy images of tibial bone and tail tendons of 14 week old *oim/oim* (B6C3fe-a/acolla2^*oim/oim*^) mouse model of OI, known to have disoriented and disorganized fibers (Hedjazi et al., 2022; Blouin et al., 2019; Nadiarnykh et al., 2007; Fratzl et al., 1996), bone fragility (Carriero et al., 2014a; Carriero et al., 2014b; Carriero et al., 2014c), and joint and tendon hyper-elasticity (Chipman et al., 1993; Enderli et al., 2016). Tissues from their wild type (WT) counterparts were used as healthy control references of well aligned, continuous fibers within tissues. The *oim* mouse model of OI presents with a naturally occurring collagen type I mutation and similar phenotypic expressions to humans with moderate to severe OI (Chipman et al., 1993; Enderli et al., 2016). Here, we are using *oim* bone and tendon tissue as a model of disorganized collagen fibers as previously observed using polarized light microscopy, SHG microscopy (Hedjazi et al., 2022; Blouin et al., 2019; Nadiarnykh et al., 2007) and small angle X-ray scattering (Fratzl et al., 1996). Yet these observations remained qualitative in nature (Blouin et al., 2019).

To prepare the bone samples, mouse tibiae transverse or longitudinal cross-sections (3 mm thick) at approximately 37% mid-diaphyseal tibial length were cut using a slow speed saw machine with a diamond blade, glued on microscope slides, and polished starting with 400 grit silicon carbide paper to a progressively higher finish until a final polishing with 0.5 µm diamond suspension solution. To prepare the tendon samples, mouse tails were excised from the body and the skin removed to expose the tail tendon. Bone and tendon samples were imaged using a Prairie Tech. Ultima IV Multiphoton Microscope (Bruker; Madison, WI) while maintained hydrated in saline solution (PBS). The microscope was equipped with a commercial titanium sapphire femtosecond two-photon laser tuned at 920 nm excitation wavelength to achieve high energies sufficient for a SHG signal and allow for optimal birefringence. The 460/50 nm bandpass filter used to detect emitted signals granted a spectral window between 410 nm and 510 nm. Backward SHG microscopy images were obtained from the transversely cut sections using a 40 × 0.8 N.A. water immersion objective lens. Images acquired were of three possible resolutions, (512 × 512, or 1024 × 1024 or 2048 × 2048 pixels) with a pixel size of 0.11 μm. Frame averaging was set at four and dwell time per pixel was defined as 0.8 s.

### FiberO algorithm

**FIGURE 1:**
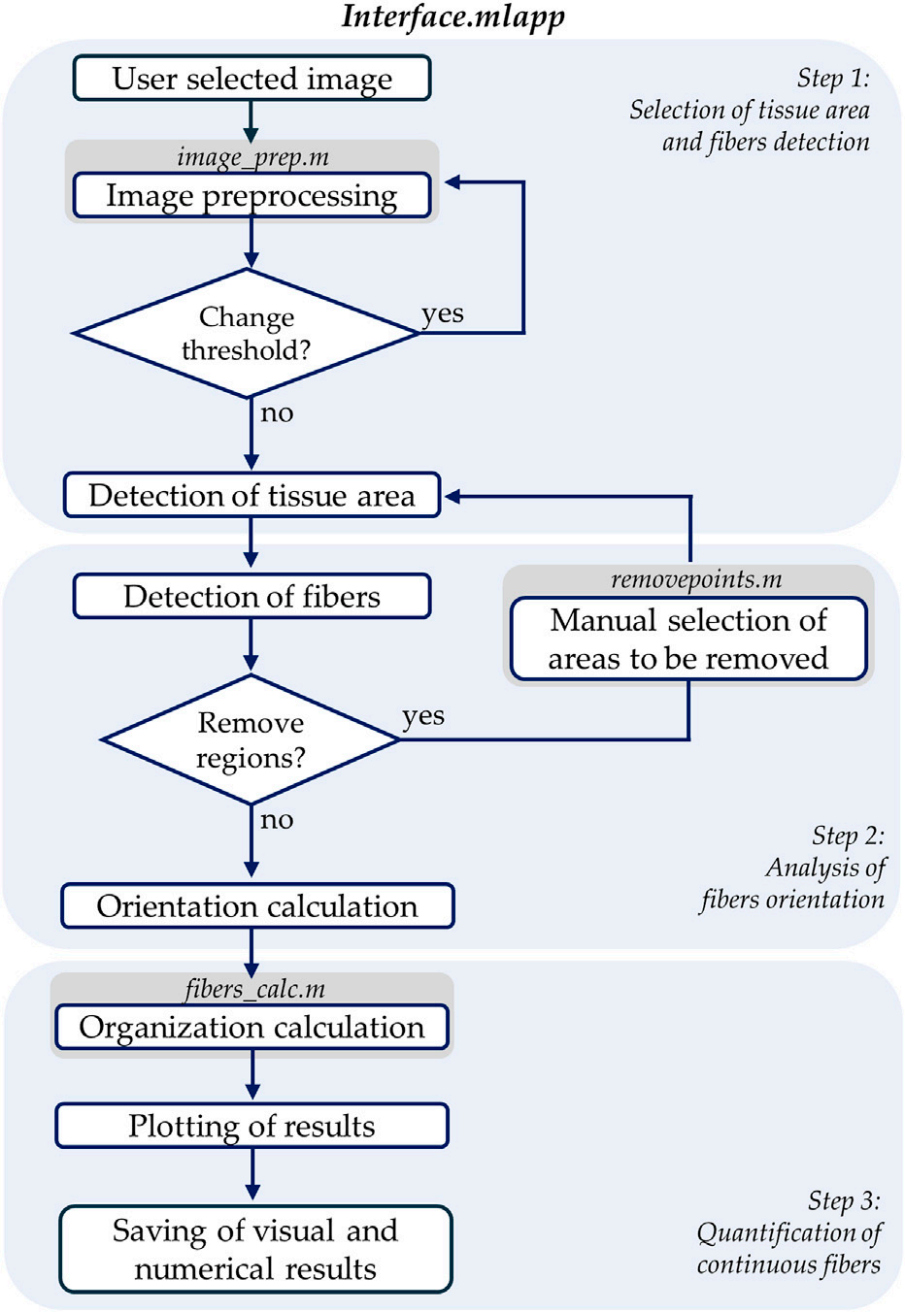
Flowchart of the main interface file containing the primary code of *FiberO*. The flowchart is divided into three main steps where the different actions and respective MATLAB scripts are included.

We created a custom MATLAB algorithm with a user interface, called *FiberO*, to automatically identify and measure the orientation of fibers as well as the quantity of organized fibers in images of fibrous tissues (Figure 1). The software is available on GitHub for public access and use in the following link: https://github.com/CarrieroLab/FiberO.

#### Step 1: selection of tissue area and fibers detection

**FIGURE 2:**
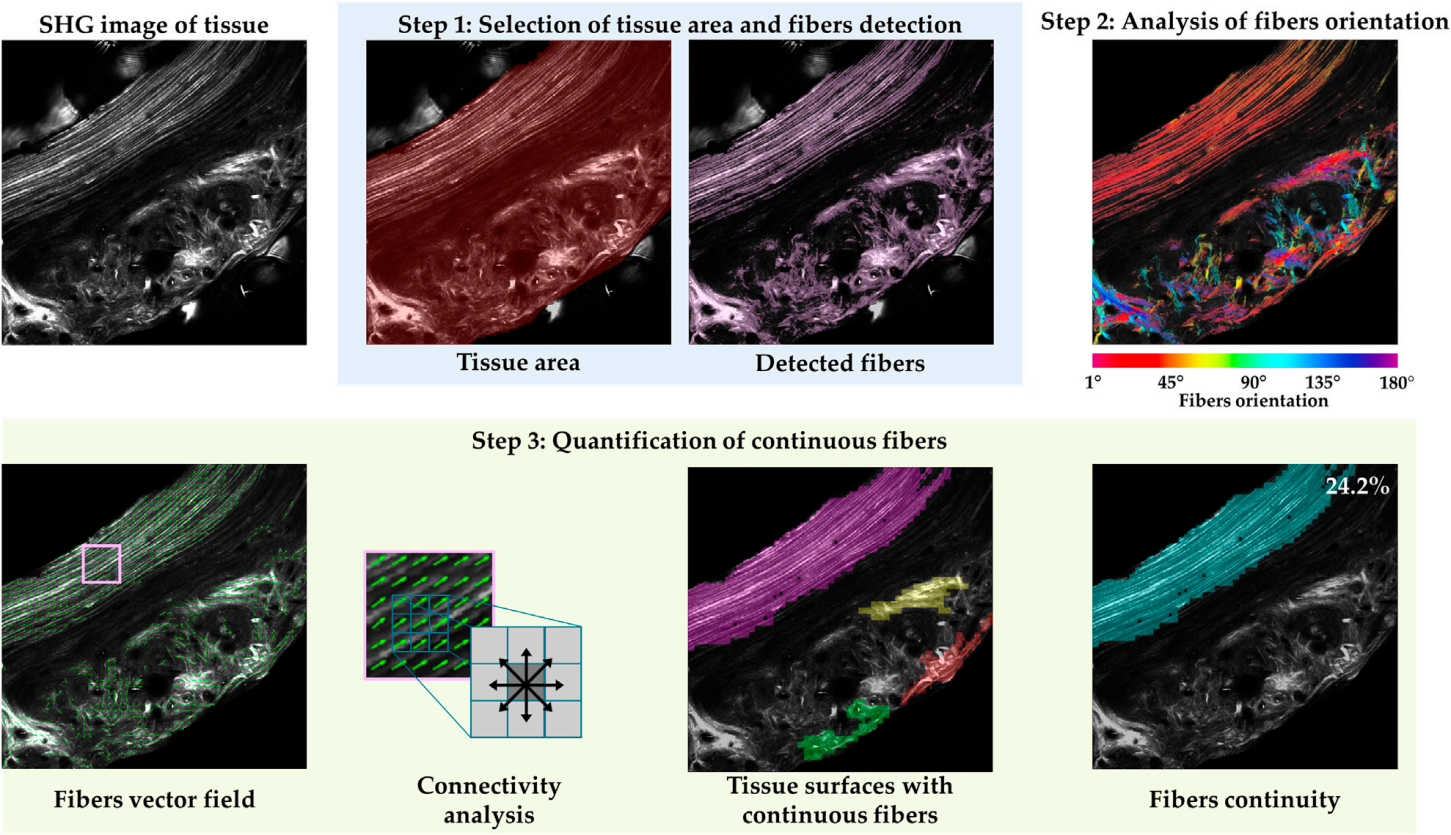
Pipeline of *FiberO* methodology illustrating the various stages for a representative SHG image of a transverse section from an *oim* mouse bone. Step 1 involves selecting the image of interest by removing artifacts and background. The bone image reveals two background areas without any information. In Step 2, the fibers orientation in the detected fibers area is calculated using a local morphological image opening. Fibers at 0° and 180° are oriented horizontally. Step 3 quantifies the fibers continuity within this tissue, and surfaces with continuous fibers in the image are based on the regional principal orientation specified by the vector fields, and subsequently only those surfaces with an area higher than 1/6 of the detected tissue area are included in the final fibers continuity density and spatial mapping.

SHG microscopy can effectively capture collagen-specific images of bone sections or tendon segments, but these images can also be characterized by low signal-to-noise ratio in areas with little collagen, or in the background, and the appearance of artifacts, such as those arising from laser power fluctuations, imaging artifacts and possible air bubbles forming while imaging in wet conditions. Therefore, the first step of the methodology is to remove the unwanted pixels of background and artifacts (e.g., air bubbles in SHG images) from the images with an automatic threshold that determines the minimum intensity value of a pixel to proceed further with the analysis (Figure 2 Step 1). Additionally, a visual inspection can be performed, allowing the user to manually select and remove remaining background and artifacts pixels in the raw images by clicking on various points to define the vertices of a polygonal area for elimination. Next, the images are divided in a total of 100 by 100 facets (which size in pixels depends on the size of our images, e.g., 50 × 50 pixels for 512 × 512 pixel images, 100 × 100 pixels for 1024 × 1024 pixel images, and 200 × 200 pixels for 2048 × 2048 pixel images). At this point, facets with noisy data were assigned as NaN values and were not considered for the next processing steps. This denoising step allows for a boost in the performance of the custom written code and a reduction in the overall computational time. If artifact areas are within the tissue, they count as part of total tissue area of interest; however, if they are connected with the edge of the image, they do not count towards to the total tissue area of interest. Besides this, artifact areas do not interfere with subsequent analysis steps.

#### Step 2: analysis of fibers orientation

Firstly, the detection of fibers is necessary to identify which pixels should be considered for the orientation calculation. To achieve this, image sharpening is applied to enhance fine details and edges within the image. This process utilizes gradients in pixel intensities along the horizontal and vertical directions to identify areas where fibers are likely to be present, making them more distinguishable for subsequent analysis (Joshi and Koju, 2012). After computing the gradients, adaptive thresholding is applied, which takes into account the intensity variations within smaller regions, or neighborhoods, of the image. This adaptive approach helps overcome challenges existing in some type of imaging, such as SHG microscopy, including issues like non-uniform illumination or varying fiber intensities. After these processes, fibers located in the foreground of the image are differentiated from the dark background of the image. However, it is important to note that some of the detected patterns might not be bright enough to be considered as collagen fibers. To ensure precise detection of fibers a final intensity threshold is applied. At this point the code calculates *the fibers density* (quantity of the fibers in the tissue) and *the fibers average intensity* (quality of the fibers in the tissue), and assesses *the fibers orientation* in the tissue.

To assess the orientation of fibers, a mathematical morphology technique with an opening of an image is implemented to erode away the boundaries of regions of foreground pixels, followed by a dilation to add pixels to the boundaries of objects in images (Figure 2 Step 2). By applying them sequentially, foreground regions that match the shape of the structuring element used in the operation can be preserved, while all other pixel regions are removed. At this point, a structural element with the shape of a line, which assimilates to the shape of fibers, is rotated starting from an angle of 1° up to 180°, and for every pixel position, the orientation in which the output image has the most intense value is saved. Obtaining the orientation for every pixel, the fibers orientation colormaps are generated (Figure 2 Step 2). These images show the direction of the fibers in the entire tissue section.

#### Step 3: quantification of continuous fibers

To determine how the fibers are organized in the tissue, *the fibers continuity* is assessed. As a first step, the vector field indicating the regional preferential orientation of the fibers is generated. For this step, the images are again divided in a grid of 50 x 50 facets. Because the images are divided in a grid of facets, the mode or the most frequent orientation value for all the pixels inside every facet is calculated, representing the main orientation of the fibers within each facet (Figure 2 Step 3 Fibers vector field). Pixels with very low intensity values and a preferential orientation of 180° are not accurately characterized by the morphological opening, significantly affecting the direction of the vectors in the vector field. As a result, they are excluded from further consideration. Next, we performed the analysis of tissue surfaces with continuous fibers by considering the eight neighbors’ connectivity for every facet (Figure 2 Step 3 Connectivity analysis). In this process, each facet is considered to be connected to the adjoining facets if the difference of their principal orientation is smaller or equal to 20°. Doing this, the whole set of surfaces that compose the bone sections are obtained. Only groups of continuous fibers whose surfaces covered more than 30 facets (over the total 50 × 50 facets) are displayed. Next, the surfaces are sorted in descending order based on of their overall surface area (Figure 2 Step 3 Tissue surfaces with continuous fibers), and only those surfaces with an area bigger than a sixth of the firstly detected fibers surface area in the analyzed image are maintained and considered as surfaces with continuous well-oriented fibers (Figure 2 Step 3 Fibers continuity). Finally, the percentage of tissue with continuous fibers is obtained as the ratio between the sum of the surfaces with continuous fibers and the detected tissue area.

#### Validation

The precision of *FiberO* in determining fibers orientation was verified by comparing it with various well-established techniques typically employed for analyzing fiber networks. A dataset of 75 images featuring artificial fiber networks was created for this validation (Supplementary Figures 1, 2), as previously done in the literature (Morrill et al., 2016). These images contained 120 lines each representing a different fibers configuration and were divided into two distinct groups to understand the accuracy of our technique when working with images containing different fiber sizes and degree of isotropy. The first subset of images consisted of 25 images featuring three different fiber widths (0.5, 1, and 1.5). The fibers were randomly arranged, with 70% (or α = 0.7) aligned in the preferred direction. The second subset of images contained 50 images with fibers that were arranged in random orientations with varying values of isotropy (α = 0.2, 0.4, 0.6, 0.8, and 1) and had a fiber width of 1.

The performance of *FiberO* was evaluated against *Directionality* (Liu, 1991) and *OrientationJ* (Püspöki et al., 2016), two plugins available in ImageJ, as well as *FiberFit* (Morrill et al., 2016) and *CT-FIRE* (Bredfeldt et al., 2014), standalone tools developed by researchers for analyzing fibrous images. *Directionality* offers two different solutions: the first one is based on Fourier spectrum analysis and the second one is based on the derivation of the local gradient orientation (Liu, 1991). *OrientationJ* computes the orientations based on the gradient structure tensor in a local neighborhood (Püspöki et al., 2016). *FiberFit* utilizes the Fourier transform to generate fiber angle distributions (Morrill et al., 2016), while *CT-FIRE* employs the fast discrete curvelet transform for image denoising and fiber edge enhancement, followed by the *FIRE* fiber extraction algorithm, enabling the precise identification and analysis of individual fibers in complex fibrous tissues (Bredfeldt et al., 2014). The errors between the measured and real principal angle, and between the measured and real amount of fibers oriented within the preferred orientation α were analyzed. To compute α, the sum of counts from the center of the histogram (± the standard deviation of the distribution) was divided by the total counts in the histogram. Statistical analysis was conducted using SPSS (IBM v.28.0) to assess significant differences in the effectiveness of the different image analysis tools. The assumptions for parametric tests were first assessed using the Kolmogorov-Smirnov (K-S) test to evaluate normal distribution and Levene’s test to determine homogeneity of variance across the techniques. As the assumptions were not satisfied, statistical differences were evaluated using the Kruskal-Wallis test, a non-parametric alternative to ANOVA. P-values were adjusted for multiple comparisons using the modified Bonferroni correction, as the focus was on evaluating *FiberO*’s performance in relation to each individual tool, rather than comparing the rest of the tools to one another. A visual inspection of the orientation colormaps produced by four different techniques (*FiberO*, *Directionality* in both modes, and *OrientationJ*) was performed using a representative SHG microscopy image of bone to assess the effectiveness of these spatial image processing methods. *FiberFit* and *CT-FIRE* were not included as they do not provide fibers orientation map.

## Results

**FIGURE 3:**
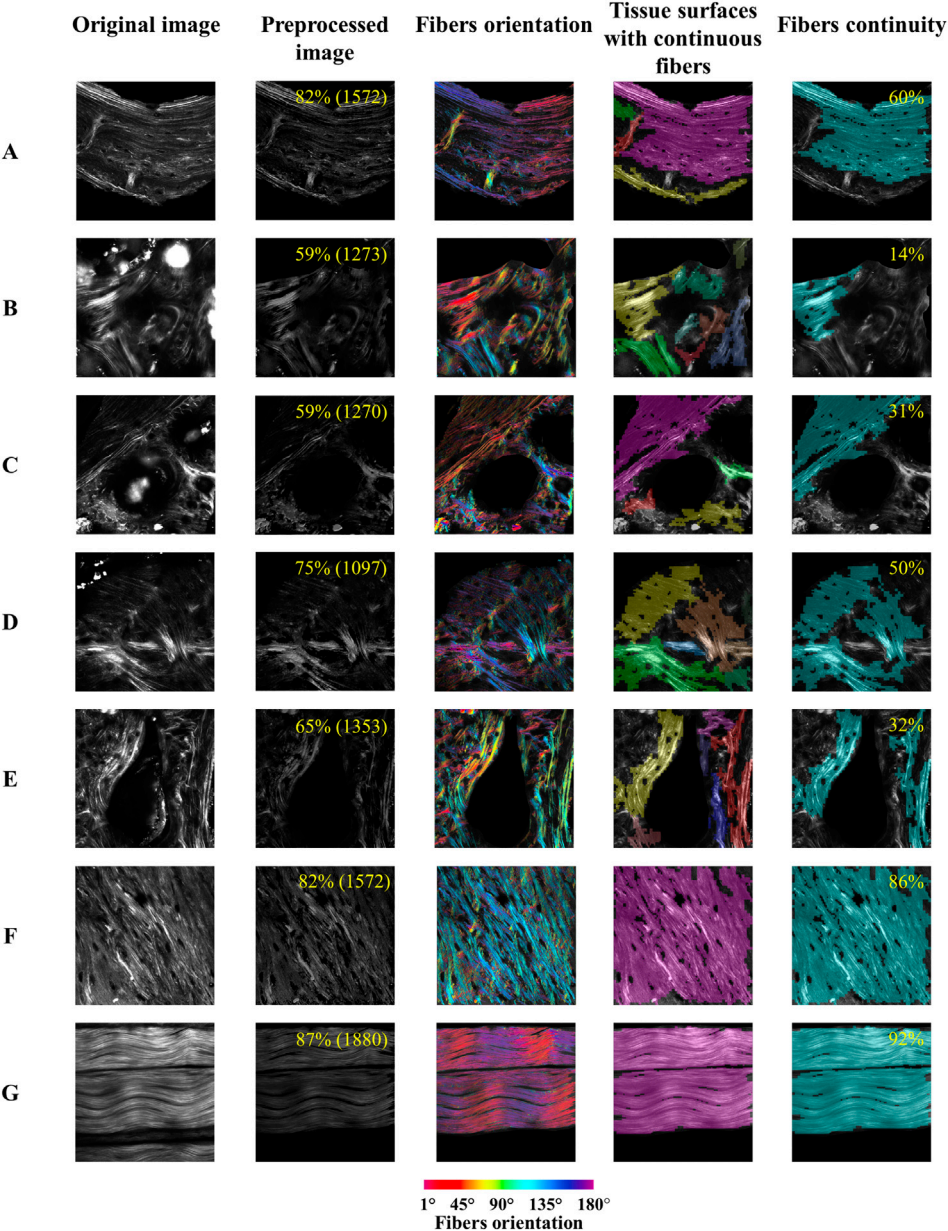
*FiberO* pipeline of results for the analysis of collagen fibers in different SHG microscopy images of bone and tendon. The results include the original image, the preprocessed image after Step 1 (indicating the fibers density and the average fibers intensity in brackets in yellow in the right upper corner), fibers orientation (with fibers at 0° and 180° oriented horizontally), tissue surfaces with continuous fibers, and fibers continuity (with the percentage of fibers continuity provided in yellow in the right upper corner). **(A)** Mouse cortical bone cross-section with organized fibers. **(B)** Mouse bone cross-section with disorganized fibers. **(C)** Mouse cortical bone cross-section with artifacts produced by water bubbles. **(D)** Knot of collagen fibers in mouse bone cross-section. **(E)** Mouse bone cross-section with a big vascular channel. **(F)** Mouse cortical bone longitudinal section. **(G)** Mouse tail tendon with three packing of undulated parallel fibers.

Here we present an automated and quantitative evaluation of fibers orientation and organization in bone and tendon SHG microscopy images as assessed by our novel *FiberO* software. Figure 3 shows the outcome of *FiberO* image processing steps for different bone and tendon SHG microscopy images. These images show the original SHG microscopy images on the first column, the preprocessed image (with fibers density and average fibers intensity) on the second column, the fibers orientation on the third column, followed by the tissue surfaces with continuous fibers, and finally by the mapping (and density) of the fibers continuity.

### Case A: mouse cortical bone cross-section with organized fibers

The sequence of images in Figure 3A exhibit a bone section mostly composed of concentrical lamellae oriented principally around the endosteum and periosteum of an *oim* mouse bone, following its natural curvature. In the output image, most of the collagen is highlighted, with some collagen fibers oriented perpendicular to the main direction of the remaining collagen fibers in the regions of blood vessels. Angiogenesis precedes osteogenesis and therefore it is interesting to visualize how the bone fibers well align around the intracortical blood vessels. Nevertheless, these continuous fibers groups are too small to be counted in the fibers continuity.

### Case B: mouse cortical bone cross-section with disorganized fibers

Figure 3B reveals a bone section with a defined preferential orientation of the collagen fibers in the left half of the image, and a more disorganized matrix in the right side of the image. *FiberO* is able to pick up these two areas automatically, and quantifies the area of collagen fibers continuity to be only 14% of the detected bone tissue area in an *oim* mouse bone.

### Case C: mouse cortical bone cross-section with artifacts produced by water bubbles

Figure 3C is characterized by a region with continuous collagen lamellae with a preferred orientation on the top, next to an area of disorganized collagen fiber tissues in an *oim* mouse bone. Two bubbles are depicted in this image and they cover large portion of the disorganized tissue area. *FiberO* here detects the artifacts and does not analyze these three parts of the image. Furthermore, *FiberO* automatically and correctly selects the area of collagen fiber continuity and estimates it to be 31% of the total bone surface in the image.

### Case D: knot of collagen fibers in mouse bone cross-section

The collagen fibers arrangement in Figure 3D shows a knot pattern in a WT mouse bone. The orientation of the fibers at the knot region seems to overlap, and are mostly well-aligned, and continuous for 50% of the entire bone surface imaged as calculated by *FiberO* software.

### Case E: mouse bone cross-section with a big vascular channel

Figure 3E presents the fiber tissue surrounding a big vascular canal of irregular shape in a WT mouse bone. In this bone 32% of the tissue fibers are continuous and mainly located around the edges of the canal, following its shape.

### Case F: mouse cortical bone longitudinal section

Figure 3F is a SHG microscopy image of a longitudinal bone section in a WT mouse cortical bone. In this image most of the collagen fibers are oriented at an angle of 120°. In the output image of *FiberO*, 86% of the fibrous surface is highlighted with continuous fibers as the low-signal facets are not considered as continuous parts.

### Case G: mouse tail tendon with three packing of undulated parallel fibers

Figure 3G shows the continuous collagen fibers of an *oim* mouse tail tendon. Fibers follow a wavy shape in a given direction. 92% of the entire tissue surface is composed by continuous fibers and is described by our software *FiberO*.

### Validation

**FIGURE 4:**
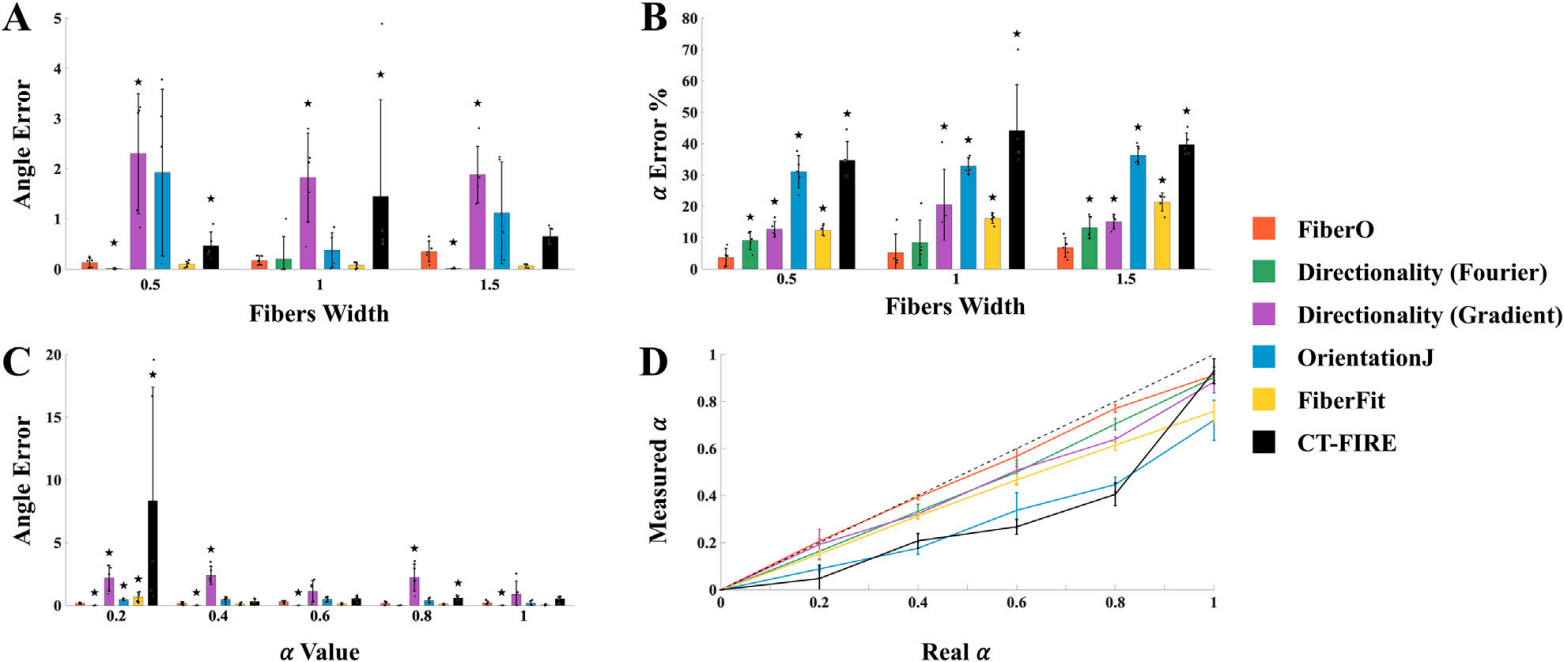
Mean and standard deviation results from the validation tests conducted using *FiberO*. **(A)** Error in the estimation of the principal orientation angle for images with varying fibers width. **(B)** Error in the calculation of the amount of fibers oriented at the principal orientation angle for images with varying fibers width. **(C)** Error in the assessment of the principal orientation angle for images with varying amount of fibers oriented at specific principal orientation angles. **(D)** Real vs. measured amount of fibers oriented at the principal orientation angle for images with varying amount of fibers oriented at the principal orientation angle. ★ denotes a statistically significant difference compared to *FiberO*, with p < 0.05.

The performance of the here developed code was analyzed comparing the results provided by *FiberO* for the analysis of new images generated with known fibers thickness and orientation (Supplementary Figures 1, 2) with the ones obtained using reliable fibers orientation determination tools: 1) *Directionality*, which computes the fiber orientations based on the Fourier spectra or the local gradient orientation (Liu, 1991), 2) *OrientationJ*, which is based on the gradient structure tensor in a local neighborhood similarly to our *FiberO* software (Püspöki et al., 2016), 3) *FiberFit*, which also employs the Fourier transform to generate fiber angle distributions (Morrill et al., 2016), and 4) *CT-FIRE*, which combines the fast discrete curvelet transform for image denoising and fiber enhancement with the FIRE algorithm for fiber extraction (Bredfeldt et al., 2014). The graphs in Figures 4A, B present the validation results for all the techniques with varying fibers width in terms of the error for the detected preferential orientation angle and the amount of fibers oriented at this specific direction α. When changing fibers width, *FiberO*’s angle error for different fiber thicknesses was only outperformed by very little (<0.2°) by the Fourier spectra-based Directionality method for fibers measuring 0.5 and 1.5 in width. In contrast, both *OrientationJ* and *CT-FIRE* performed worse than *FiberO*, exhibiting significantly higher angle errors at different fiber widths. Additionally, when evaluating the percentage error between the actual and measured number of fibers aligned at the preferential orientation α, *FiberO* outperformed all other methods, with an error rate of 5.3% ± 4.1%. It was followed by the Fourier spectra-based *Directionality* method, then the local gradient-based *Directionality* method, *FiberFit*, *OrientationJ*, and finally *CT-FIRE*, with an average error of 10.3% ± 5.0%, 16.1% ± 7.1%, 16.7% ± 4.3%, 33.4% ± 4.1%, and 39.6% ± 9.6%, respectively (Figure 4B). These results show that *Directionality*, *FiberFit*, *CT-FIRE*, and *OrientationJ* methods underestimate the amount of fibers that follow the preferential orientation of the network.

Figure 4C presents the angle error data obtained from the set of images with different values of α. Once again, the smallest error between the real and the measured preferential orientation was obtained using the Fourier spectra *Directionality* method, closely followed by *FiberO*, which achieved a significantly lower error compared to the other techniques across various levels of anisotropy, with the poorest performance observed in the local gradient-based *Directionality* method. When a high level of disorganization was present, with numerous fibers exhibiting random orientations and lacking a clear preferential direction, *CT-FIRE* was unable to produce results that accurately reflected the actual fibers arrangement. Finally, Figure 4D displays the difference between the measured and real α for the different evaluated techniques (with a mean error value of 3.4% ± 3.7%, 7.9% ± 3.9%, 9.9% ± 4.6%, 13.3% ± 7.3%, 22.9% ± 12.8%, and 24.6% ± 9.5% for *FiberO*, *Directionality* with Fourier spectra, *Directionality* with local gradient orientation, *FiberFit*, *CT-FIRE*, and *OrientationJ*, respectively) and indicates that *FiberO* best estimates the number of fibers oriented at the principal orientation angle.

**FIGURE 5:**
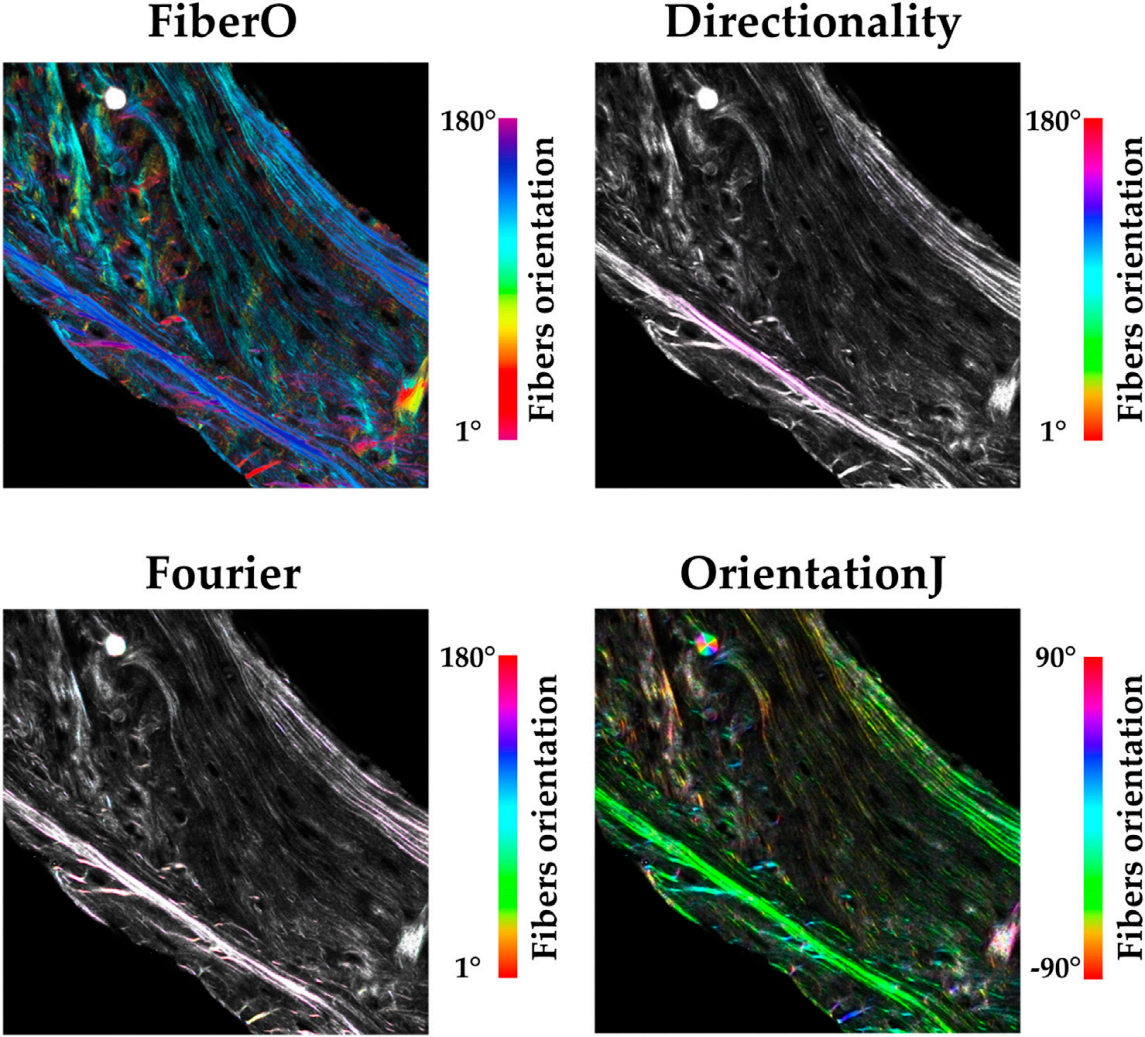
Fibers orientation colormaps obtained using *FiberO*, *Directionality* using local gradient and Fourier spectra, and *OrientationJ* for a representative bone cross-section imaged with SHG microscopy. A different color code for the fibers orientation is used for each method, accordingly to their approach. Only *FiberO* and *OrientationJ* show an intelligible distribution of the fiber orientations. Fibers are however more visible when using *FiberO*.

The orientation maps generated using the four different available techniques (*FiberO*, *Directionality* in both modes, and *OrientationJ*) for a representative SHG microscopy image of an *oim* mouse bone cross-section are shown in Figure 5. This figure shows that although the *Directionality* method based on Fourier spectra is powerful for calculating global fiber orientations (as seen in Figure 4A), it does not perform well when representing their local orientation values in the form of orientation color maps, generating images that do not show the different orientations of the fibers (Figure 5). A similar behavior is found when analyzing the results for the *Directionality* method based on the local gradient orientation, where only the orientation of the most intense fibers is highlighted by a pink-like color (Figure 5). *FiberO* and *OrientationJ* provide the visually most comprehensive fibers orientation colormaps, with detailed regional orientation of the primary orientations of the collagen fibers (Figure 5). Figure 5 also shows that in the areas with less collagen content, such as the center of the bone cortex, the intensity of the colors indicating the direction of the collagen fibers computed by *OrientationJ* is reduced compared to the colors displayed by *FiberO*, which instead clearly exhibit the regional principal orientation of the collagen fibers with the color mapping.

## Discussion

A new open-source available accurate software for the automatic and quantitative analysis of fibers orientation and organization within a tissue is presented in this work. This method, named *FiberO,* conducts the analysis of the fibers orientation and continuity in the tissue starting from gray scale images of fibers, such as those but not limited to the ones obtained using SHG microscopy imaging, which, for example, allows the visualization of collagen type I fibers within connective tissues. In biological materials, the hierarchical organization of the primary components of the extracellular matrix of connective tissues plays a crucial role in their mechanical properties. Understanding the orientation and assembly of collagen fibers in connective tissues, such as bone and tendons, is important in determining how these structures relate to the tissue mechanical properties, or are affected by disease and if therapies can improve them. This extends to other fibrous non-collagenous proteins as long as they can be visualized and distinguished from surrounding matrix via any imaging modality, not just SHG microscopy. For example, picrosirius red staining of biological tissue is another well-known histochemical technique used to study tissue fibers (Rittié, 2017). However, so far, the use of these imaging and histological techniques, and the understanding of the contribution of the fibers structure and assembly to the biological tissue mechanics has been limited by the inability to quantify fibers orientation and organization in these tissues.

The presented work provides the first open-source software that allows users to automatically and quantitatively evaluate the orientation and organization of fibers in tissues, such as in those from connective tissues, including bone and tendons. *FiberO* program is written in the high-level engineering language MATLAB and it is customizable for the users as needed. In addition, a user-friendly interface has been implemented to facilitate program control for user less experienced in coding. This allows users to specify properties and visualize the tissue sections at different steps of the analysis.

*FiberO* software was originally designed to measure bone and tendons collagen type I fibers orientation and organization, but because many living tissues are composed of extracellular fibrous proteins, it can be generalized to study other fibrous tissues, such as lung sections that are composed of elastin fibers, also visible with SHG microscopy, as well as fibers in other natural or artificial materials such as in food, rocks, engineering materials, etc.

The technique presented here has been validated against other open-source methods, which have been used for the study of fibers network orientation (Sensini et al., 2018; Reznikov et al., 2013; Fee et al., 2016; Taufalele et al., 2019), including *Directionality* (Liu, 1991), *OrientationJ* (Püspöki et al., 2016), *FiberFit* (Morrill et al., 2016), and *CT-FIRE* (Bredfeldt et al., 2014). *FiberO* is the method that most accurately determines the amount of fibers oriented at the preferential orientation angle of known fibers networks. Furthermore, *FiberO* is only outperformed by very little (<0.2°) by *Directionality* method based on the Fourier spectra in calculating the value of the preferential orientation of the fibers networks. However, although the Fourier Transform *Directionality* method proved more efficient in capturing the global information of the fiber network, it loses to localize the fibers orientation as the spatial understanding of the Fourier Transform *Directionality* method is compromised during the transition from frequency space to the spatial domain, as evidenced by the dimmed orientation maps. Therefore, when analyzing the spatial distribution of the collagen fibers network, *Directionality* method based on the Fourier spectra is not accurate and does not provide important information on the orientation, as instead is done by *FiberO*.

Besides *Directionality* and *OrientationJ*, other open-source software have been developed for the automatic calculation of fibers orientation on grayscale images. Examples of these include the software *FiberFit*, which was used to evaluate the analysis of fibers from confocal images of ligaments and only computes a single value of the global orientation distribution for the entire image, and hence does not generate the colormaps showing the local orientation of the fibers (Morrill et al., 2016). Similarly, the software *CT-FIRE* also offers the ability to measure specific characteristics of individual fibers, encompassing aspects such as orientation, size, linearity, and thickness (Bredfeldt et al., 2014), as well as notable is the work conducted in this direction by many other researchers in this field (Sivaguru et al., 2010; Bayan et al., 2009; Koch et al., 2014; Zyablitskaya et al., 2017). However, *FiberFit* and *CT-FIRE* did not demonstrate better performance than *FiberO* in the analysis of images with fibers with known width and orientation. Furthermore, because they do not provide the capability to visualize orientation maps, they are limited tools for analyzing the spatial orientation of fibers in biological tissues. Therefore, *FiberO* is to date the open-source software that best quantifies fibers orientation in the tissue with contour mapping. *FiberO* has been developed to work with 2D images. However, its implementation on stacks of images can be used to obtain the analysis of 3D images with respect of a plane of reference, as we have done previously to analyze fibers organization in tendons (Manrique et al., 2023).

To our knowledge, *FiberO* is the first and only software that based on the fibers regional organization information automatically detects the surface(s) with continuous fibers that contribute to the overall tissue imaged. This task was till now done manually by researchers to distinguish the areas of tissue with organized and disordered collagen fibers in SHG microscopy images of bone (Hedjazi et al., 2022). Manual selection increases the time required to process the data and possibly introduces subjectivity into the results. Instead, *FiberO* accurately differentiated region(s) in the analyzed images in an automatic and rapid manner based on the quantitative analysis of their fibers orientation and continuity.

In conclusion, we developed an open-source software, called *FiberO*, that based on morphological image openings can automatically and accurately quantify the main regional orientations of fibers and determine their organization in biological tissues from grey scale images, such as from those of bone and tendon collagen fibers taken with SHG microscopy. *FiberO*’s fibers orientation quantification and plotting outperformed five different open-source techniques currently used to track fibers orientations. *FiberO* is also currently the only open-source available software providing organization information of the fibers within the tissue. This study provides a novel tool for researchers to investigate the fibers orientation and organization in different natural and artificially made fibrous tissues.

## Data Availability

The original contributions presented in the study are included in the article/Supplementary Material and in the Git-Hub folder of *FiberO* (https://github.com/CarrieroLab/FiberO), further inquiries can be directed to the corresponding author.
