# Supplementary material for: FiberO for an automated quantitative analysis of fibers orientation and organization in biological fibrous tissues
Source: Front Bioeng Biotechnol. 2025 Jan 6;12:1497837. doi: 10.3389/fbioe.2024.1497837 (PMC11743555; doi:10.3389/fbioe.2024.1497837)
Supplement: Supplementary file 1 [file DataSheet1.pdf]

Supplemental material

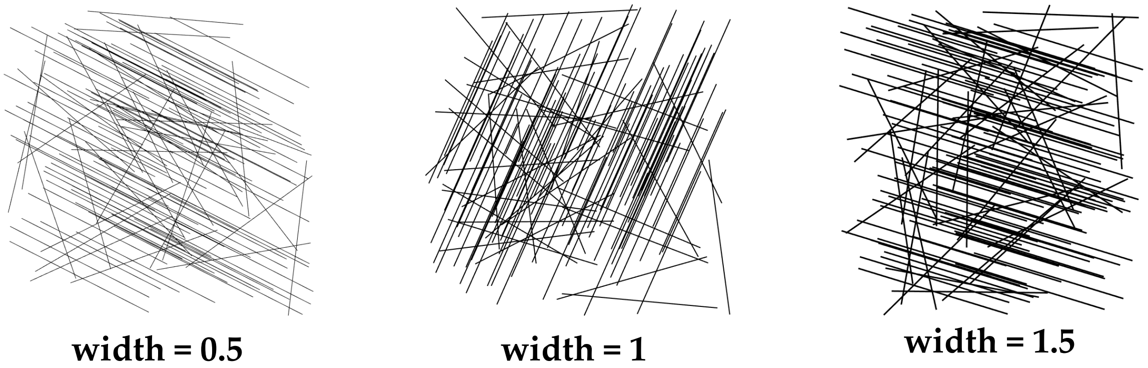

Supplementary Figure 1: Instances of images from the first dataset employed to validate the customized code are illustrated here. The initial set of images comprised 25 pictures featuring three distinct fiber width dimensions (0.5, 1, and 1.5). These fibers were randomly oriented, with 70% of them aligned in the preferred orientation (or $\alpha = 0.7$).

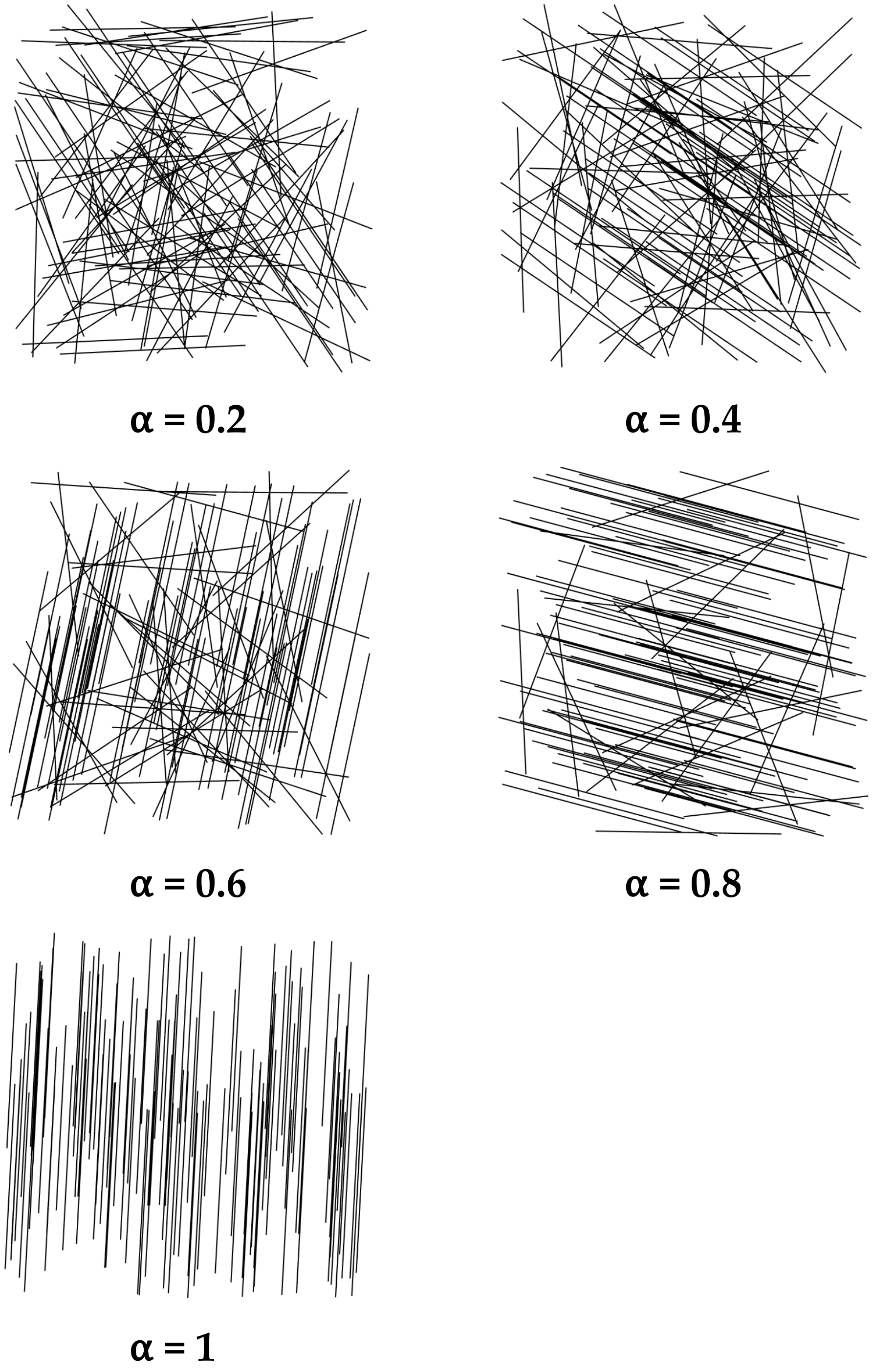

Supplementary Figure 2: Instances of images from the second dataset employed to validate the customized code are illustrated here. This subgroup comprises 50 images featuring fibers arranged in random orientations, exhibiting diverse isotropy values (α = 0.2, 0.4, 0.6, 0.8, and 1), with a consistent fiber width of 1.
